# The Psychometric Properties and Cutoff Score of the Child and Adolescent Mindfulness Measure (CAMM) in Chinese Primary School Students

**DOI:** 10.3390/children9040499

**Published:** 2022-04-02

**Authors:** Xin Chen, Kaixin Liang, Liuyue Huang, Wenlong Mu, Wenjing Dong, Shiyun Chen, Sitong Chen, Xinli Chi

**Affiliations:** 1 School of Psychology, Shenzhen University, Shenzhen 518061, China; ccchenxin19@163.com (X.C.); liangkaixin2020@email.szu.edu.cn (K.L.); 1910481006@email.szu.edu.cn (L.H.); dongwj1997@126.com (W.D.); 2 Center for Mental Health, Shenzhen University, Shenzhen 518061, China; 3 School of Economics and Management, Wuhan University, Wuhan 430072, China; mu.w@whu.edu.cn; 4 Department of Psychology and Human Development, Institute of Education, University College London, London WC1E 6BT, UK; shiyun.chen.21@ucl.ac.uk; 5 Institute for Health and Sport, Victoria University, Melbourne 8001, Australia; sitong.chen@live.vu.edu.au

**Keywords:** Child and Adolescent Mindfulness Measure (CAMM), reliability, validity, cutoff, primary school students, Chinese

## Abstract

To date, the Child and Adolescent Mindfulness Measure (CAMM) has been translated into several languages, including Chinese. This study aimed to explore the reliability and validity of the Chinese version of the CAMM and to identify the appropriate cutoff score among Chinese primary school students. A total of 1283 participants (52.2% males; 11.52 ± 0.78 years of age) completed a series of questionnaires to evaluate their mental health, including mindfulness, subjective well-being, positive youth development (PYD), depression, and anxiety. Item analysis, Confirmatory Factor Analysis (CFA), Exploratory Structural Equation Modeling (ESEM), criterion-related validity analysis, Receiver Operating Characteristic (ROC) analysis, and reliability analysis were performed. The results show that the Chinese version of the CAMM had acceptable item–scale correlation (r = 0.405–0.775, *p* < 0.001) and was the best fit for the two-factor ESEM model (*χ*^2^ = 168.251, *p* < 0.001, *df* = 26, TLI = 0.910, CFI = 0.948, RMSEA = 0.065, SRMR = 0.033) among Chinese primary school students. Additionally, the total score of the Chinese version of the CAMM was significantly associated with subjective well-being and PYD (r = 0.287–0.381, *p* < 0.001), and negatively associated with depression, and anxiety (r = −0.612–−0.542, *p* < 0.001). Moreover, a cutoff score of 22 or higher revealed a significant predictive power for all the included criteria. Finally, the Chinese version of the CAMM had good internal consistency (Cronbach’s α = 0.826, McDonald’s ω = 0.826). Altogether, the Chinese version of the CAMM had satisfactory psychometric properties, and it can be applied to Chinese children.

## 1. Introduction

Mindfulness, an important predictor of people’s physical and mental health, has received a considerable amount of attention from researchers, practitioners, and the general public in the last 10 years. In the Buddhist scriptures, mindfulness is written in Pali as sammā sati, which means maintaining a clear and proper awareness of goals in the present moment [1]. As the concept of mindfulness has been gradually introduced into the field of psychology, Kabat-Zinn [2] described it as an awareness that emerges from paying attention to the present moment in a conscious and non-judgmental way. Baer [3] noted that mindfulness is a psychological process that observes the ongoing streams of internal and external stimuli without judgment. Based on these descriptive definitions, the current study defines mindfulness as perceiving and accepting the present moment without judgment.

The development of research on mindfulness has elicited the need to identify tools to measure it. The Child and Adolescent Mindfulness Measure (CAMM) [4] is one of the few available tools for measuring mindfulness in children and adolescents, including awareness of the present moment and a non-judgmental, non-avoidant stance toward thoughts and feelings. The CAMM, a 10-item scale, is applicable to children and adolescents ranging in age from 10 to 17, and it has been validated and used in children and adolescents in many countries, such as The Netherlands [5], Australia [6], Spain [7,8], Italy [9], Canada [10], Turkey [11], Chile [8], France [12], Iran [13], Greece [14], and China [15]. The reliability and validity of the Chinese version of the CAMM was found to be satisfactory among junior high school students [15]. However, the scale has not been validated in primary school students whose cognitive and emotion regulation capabilities are different from those of junior high school students; thus, the differences in the characteristics of these types of students may lead to differences in the mindfulness measurements. As the social attention on children’s mental health increases, it is urgent to test the applicability of the Chinese version of the CAMM in primary school students to provide a simplified and effective tool in order to promote mindfulness-related research in Chinese children.

The findings from most CAMM studies conducted in other countries are consistent with the result from the original study conducted in an English-speaking population, which concluded that the CAMM consists of a reliable single factor. However, the Chinese version of the CAMM displayed a two-factor structure among Chinese middle school students; those factors are awareness and non-judgment (observing the present without judgment), and acceptance (accepting all the thoughts and feelings that arise) [15]. To examine the construct validity of the scale, most CAMM validation studies adopted Exploratory Factor Analysis (EFA) and Confirmatory Factor Analysis (CFA) for the psychometric analysis. It is important to note that the use of Exploratory Structural Equation Modeling (ESEM) is limited. The ESEM framework, which allows items to load on multiple factors, can be used in both an exploratory and confirmatory manner [16] to adequately consider more possible models. Therefore, the current study established one/two-factor models in CFA/ESEM frameworks to provide more evidence for the examination of the construct validity of the Chinese version of the CAMM.

Previous studies have shown that mindfulness is associated with positive outcomes among children, such as self-regulation of emotions [17,18], subjective well-being [19], psychological resilience [20], prosociality behaviors and empathy [21,22], and interpersonal relationships [18]. Mindfulness is also related to better concentration [17,23,24], cognitive flexibility [25], and academic outcomes [21,23,24]. Furthermore, mindfulness is connected with fewer children’s ruminations and intrusive thoughts [18], depression and anxiety [17,24], physical and verbal aggression, and other problem behaviors [23,26,27,28]. Hence, both negative and positive criteria were used to examine the criterion-related validity of the Chinese version of the CAMM.

Additionally, despite widespread use of the CAMM scale worldwide, to date, no optimal cutoff score has been proposed. Obtaining a cutoff score for the scale makes it easier to classify the participants into either a high level of mindfulness or a low level of mindfulness. It may also facilitate the ability to interpret and compare the research outcomes, thus increasing the opportunities to further explore cultural differences between different populations.

The current study aimed to examine the construct validity, criterion-related validity, cutoff score, and internal consistency of the Chinese version of the CAMM in primary school students in China.

## 2. Methods

### 2.1. Study Participants and Procedure

With the support of the Educational Science Research Institute of Shenzhen, the current study was conducted in Shenzhen, China in March 2021. The targeted participants were grade 5 and grade 6 students from 8 primary schools, who had the ability to read and understand Chinese well and were competent to finish a series of online questionnaires. Before collecting the data, all the participants and their guardians were informed of the main purpose of the study. The students who disagreed with participating in the survey and those whose guardians or teachers disagreed with them participating in the survey were excluded. With the assistance of teachers and school staff in the local schools, and with the class as a unit, participants got together to complete the online questionnaires anonymously in computer rooms, which took about 20 min. The questionnaires that were not submitted within the allotted time, were not complete, or gave excessive repetitive responses were eliminated.

### 2.2. Measurement

#### 2.2.1. Mindfulness

The Chinese version of the CAMM was used to assess each individual’s level of mindfulness. This instrument consists of 10 items assessed on a 5-point Likert scale ranging from 0 (never) to 4 (always). All items are scored in reverse, with higher total scores indicating higher levels of mindfulness. The instrument was validated and administered to Chinese youth in previous research [15].

#### 2.2.2. Subject Well-Being

The World Health Organization—Five Well-being Index (WHO-5) uses 5 items to measure children’s subjective well-being [29]. Each item is assessed on a 6-point Likert scale ranging from 0 (none) to 5 (always), with higher total scores indicating higher levels of subjective well-being.

#### 2.2.3. Positive Youth Development (PYD)

This study used the Five Cs of Positive Youth Development—Very Short Form (PYD-VSF) to measure PYD [30]. The adapted 16-item Chinese version of the PYD-VSF has been demonstrated to have acceptable reliability and validity in Chinese youth [31]. Each item was rated on a 5-point Likert scale from 1 (not at all) to 5 (very much), with higher total scores indicating better positive development.

#### 2.2.4. Depression Symptoms

Depression symptoms were measured using the Chinese version of the 9-item Patient Health Questionnaire (PHQ-9). This instrument consists of 9 items assessed on a 4-point Likert scale ranging from 0 (never) to 3 (nearly every day), with higher total scores reflecting more severe depression symptoms. The severity of depression symptoms can be classified based on the total PHQ-9 scores: 0–4, minimal; 5–9, mild; 10–14, moderate; 15–19, moderately severe; and 20–27, severe. Previous studies show that the Chinese PHQ-9 version is appropriate to Chinese youth [32,33].

#### 2.2.5. Anxiety Symptoms

The 7-item Generalized Anxiety Disorder Scale (GAD-7) can be used to measure anxiety symptoms [34]. The Chinese version of the GAD-7 has been validated and used in the Chinese population [35]. It consists of seven items, each of which is rated on a 4-point Likert scale from 0 (not at all) to 3 (nearly every day), with a higher total score indicating more severe anxiety symptoms. The severity of anxiety can be classified as minimal (0–4), mild (5–9), moderate (10–14), and severe (15–21).

### 2.3. Statistical Analyses

First, the total score data of the Chinese version of the CAMM were used for item analysis in SPSS version 26.0 software, including item–total correlation and the independent samples T-test for the high-score group and the low-score group (both were 27%).

Second, the construct validity of the Chinese version of the CAMM was examined. The maximum likelihood (ML) estimation was used for the Kaiser-Meyer-Olkin (KMO) test and the Bartlett’s test to ensure the feasibility of the factor analysis. According to previous studies, the data were used to establish one-factor models and two-factor models in the CFA and ESEM frameworks, which were run via Mplus version 8.3 software with robust maximum likelihood (MLR) estimation and target oblique rotation. In the ESEM models, cross-loadings were allowed but they tended to be zero [36]. The two-factor models were established based on existing research in China [15]. Factor 1 is awareness and non-judgment, including items 1, 2, 3, 6, 7, and 8. Factor 2 is acceptance, including items 4, 5, 9, and 10. The best model was then selected based on the chi-square test value, the degree of freedom, and several model fit indices: Root Mean Square Error of Approximation (RMSEA), Bentler’s Comparative Fit Index (CFI), the Tucker–Lewis Index (TLI), and standardized root mean square residual (SRMR). For an adequate model fit, the indices’ criteria should meet the CFI and the TLI > 0.90, and RMSEA and SRMR < 0.05, with <0.08 being satisfactory [37,38].

Third, this study estimated the coefficients of correlation between mindfulness and each criterion according to previous studies [6,7,9,11,17,19,24] to test the criterion validity, particularly, subjective well-being, PYD, depression, and anxiety.

Fourth, Receiver Operating Characteristic (ROC) analysis was performed to define the appropriate cutoff score for the Chinese version of the CAMM in relation to the abovementioned variables, which served as the external criteria. Dichotomous variables were created out of the total WHO-5, PHQ-9, and GAD-7 scores, using the cutoff score of 10 to assess subjective well-being, depression, and anxiety, respectively [29,32,35]. Moreover, according to the mean total score, the participants were categorized based on the cutoff score of 60 for PYD. After identifying the cutoff points, the participants with a total score above the given cutoff value were considered to be cases with a high level of mindfulness. Those with a total score below the given cutoff value were regarded as having a low level of mindfulness. The Youden index was used to determine the optimal cutoff score and to reduce the risk of misclassification.

Finally, the reliability of the scale was examined by its internal consistency, indicated by Cronbach’s α and McDonald’s ω.

## 3. Results

### 3.1. Participant Characteristics and Reliabilities of Measurements

In total, 1584 students initially received the survey invitation and 131 students refused to participate in the current study. After excluding invalid data, the final sample consisted of 1283 children aged 10–14 years (mean age = 11.52 years, SD = 0.78). Participant information is detailed in Table 1, including gender (male 52.2%, female 47.8%); grade (grade 5 50.3%, grade 6 49.7%); and sibling, paternal, and maternal education. The reliabilities of the WHO-5, PYD-VSF, PHQ-9, and GAD-7 in the current study were greater than 0.900.

### 3.2. Item Analysis

The item–scale correlation coefficient ranged from 0.405 to 0.775 (*p* < 0.001), which is greater than 0.400. Moreover, there was a significant difference between the high-score and low-score groups (*p* < 0.001). Therefore, all 10 items were retained. Independent sample T-test results showed that there was no difference in the level of mindfulness between males and females (*p* = 0.626), or between grade 5 and grade 6 (*p* = 0.492).

### 3.3. Construct Validity

**Figure 1:**
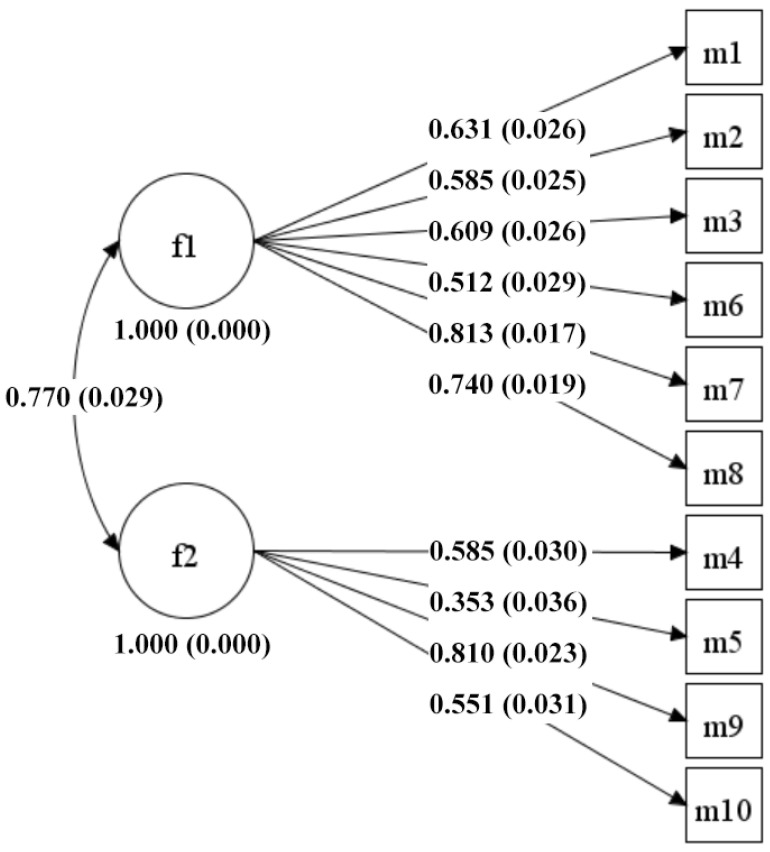
Diagrams of the two-factor CFA standardized model of the Chinese version of the CAMM for primary school students (*n* = 1283). f1 and f2 are the two factors of the Chinese version of the CAMM; m1−m10 are items 1−10.

**Figure 2:**
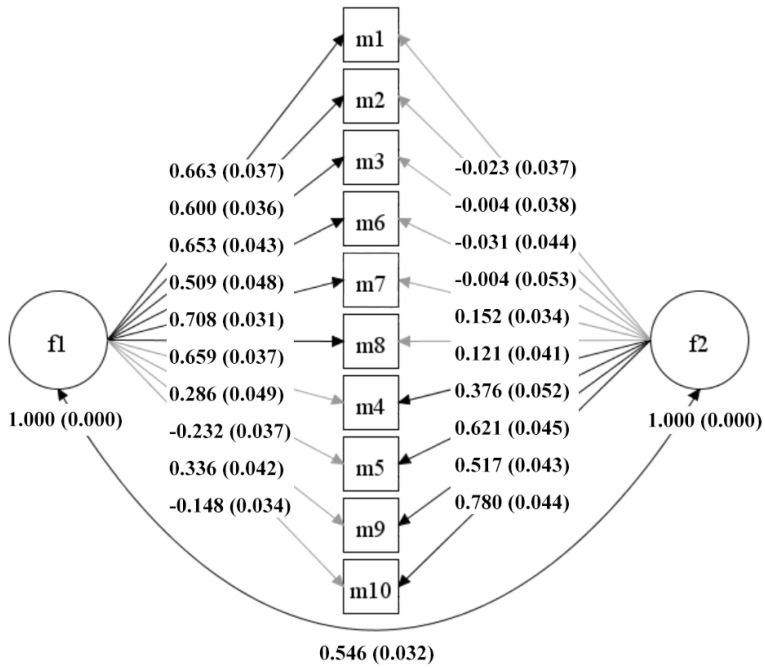
Diagrams of the two-factor ESEM standardized model of the Chinese version of the CAMM for primary school students (*n* = 1283).

The KMO value of the data was 0.877 (*p* < 0.001), and the value of the Bartlett’s test was 3966.650 (*p* < 0.001), which indicated the feasibility of factor analysis. There were two factors that showed initial eigenvalues greater than 1, specifically 4.090, and 1.356. The variance rates were 40.896% and 13.561%, and the cumulative variance rate was 54.457%. The CFA and ESEM results indicated that (see Table 2), in comparison to the one-factor model, the two-factor model had a better imitative effect for the Chinese version of the CAMM regardless of which frameworks were used. The model fit indices of the two-factor ESEM model were superior to those of the two-factor CFA model, presenting a preferable psychometric quality in both the previous Chinese study [15] and the current study. The standardized factor loadings of the two-factor CFA model and the two-factor ESEM model are shown in Figure 1 and Figure 2, respectively. The factor loading of the two-factor ESEM model ranged from 0.376 to 0.780, and the correlation coefficient of the two factors is 0.546 (*p* < 0.001), which is lower than that of the two-factor CFA model. Therefore, the Chinese version of the CAMM was the best fit for the two-factor ESEM model, with satisfactory construct validity among Chinese primary school students.

### 3.4. Criterion-Related Validity

In this study, subjective well-being, PYD, depression, and anxiety were used as the criteria. As shown in Table 3, after controlling for gender and grade, the total score and factor scores of the Chinese version of the CAMM were significantly positively correlated with subjective well-being and PYD; they were significantly negatively correlated with depression and anxiety. These findings indicate that the scale had an acceptable criterion-related validity.

### 3.5. Cutoff Score

To identify the appropriate cutoff score of the Chinese version of the CAMM, the ROC curve and Youden index were used to determine the predictive validity of the scale for subjective well-being, PYD, depression, and anxiety. The value of the Youden index provided the best tradeoff between sensitivity and specificity [39]. According to the Youden index values presented in Table 4, a cutoff score of 22 or higher was optimal for the children in the current study.

### 3.6. Internal Consistency

The internal consistency of the Chinese version of the CAMM among primary school students was indicated by Cronbach’s α and McDonald’s ω. The Cronbach’s α of the scale had a value of 0.826; the Cronbach’s α values for factor 1 and factor 2 were 0.815 and 0.689, respectively. The McDonald’s ω of the scale had a value of 0.826; the McDonald’s ω was 0.707 for both factor 1 and factor 2.

## 4. Discussion

Overall, this study explored the reliability and validity of the Chinese version of the CAMM in Chinese primary school students so as to enrich the tools used to measure mindfulness in China. The analytical results showed that the Chinese version of the CAMM had acceptable item–scale correlation and satisfactory discrimination. The two-factor ESEM model had the best fit indexes among the Chinese primary school students. After controlling for gender and grade, the scores of the scale were significantly positively correlated with subjective well-being and PYD; they were significantly negatively correlated with depression and anxiety. The optimal cutoff score of the Chinese version of the CAMM was 22 or higher for children. The scale also had good internal consistency and composite reliability. In summary, the Chinese version of the CAMM has satisfactory psychometric quality and it can be applied to Chinese children to measure the level of mindfulness.

Specifically, different from the results obtained from studies conducted in other countries, the Chinese version of the CAMM was more aligned with the two-factor model than the one-factor model for both the CFA and ESEM frameworks. To explain the difference, the original version of the CAMM was adapted from three of the four facets found on the Kentucky Inventory of Mindfulness Skills [40], namely, assessing mindfulness in the dimensions of observing, acting with awareness, and accepting without judgment [6]. These dimensions are similar to the two dimensions of the Chinese version of the CAMM: “awareness and non-judgment” and “acceptance”. Additionally, the descriptive definitions of mindfulness [4,5] commonly emphasized non-judgment and acceptance [41]. To measure non-judgmental acceptance, operational definitions of mindfulness were proposed. Bishop et al. [42] regarded mindfulness as a state-like quality containing two dimensions: self-regulation to attention and orientation to one’s experience. Thus, for both the descriptive definition and the operational definition, the concept of mindfulness has a two-dimensional structure that is similar to the two dimensions of the Chinese version of the CAMM. Furthermore, the results may also reveal a cultural difference. Compared with people in other countries, the Chinese people attach more importance to academic performances, so children have to avoid distractions and focus on their studies. Thus, acceptance is important in the Chinese context and has become an independent dimension of the Chinese version of the CAMM. The Dutch version of the CAMM [5] and the Persian version of the CAMM [13] also found two-factor structures, and their factor names were similar to those in the Chinese version of the CAMM. The two dimensions of the Dutch version of the CAMM are “present moment awareness” and “avoidance of thoughts and feelings”. The two dimensions of the Persian version of the CAMM are “present-moment non-judgmental awareness” and “suppressing or avoiding thoughts and feelings”. Consequently, the two-dimensional structure result obtained in the current study is acceptable.

It is worth noting that the current study’s result indicated that the Chinese version of the CAMM had better goodness of fit when using the ESEM than when using the CFA. The ESEM models showed a better imitative effect than that of the CFA models. Given that mindfulness tends to be a multi-construct, a certain degree of association could be present between the items and the non-target, but conceptually related factors, that is, some cross-loadings between factors, should be expected. In the ESEM model, cross-loadings were allowed. In the CFA model, cross-loading was specified at zero, which was more restrictive than that in the ESEM model [16]. Thus, the ESEM framework can adequately consider more possible models, reducing biases and avoiding unsatisfactory representations of the construct [16,43,44,45].

The current study also found that there was no difference in the level of mindfulness between males and females, which is similar to previous studies [6,9,12]. Evaluation of the criterion-related validity found that, after adjusting for gender and grade, there was a higher level of mindfulness, subjective well-being, and PYD, and a lower level of depression and anxiety, which is similar to the results reported in previous studies [7,9,11]. Not only did these results validate that the scale had a satisfactory criterion-related validity, but they also indicated that mindfulness could act as a strong predictor of children’s mental health [19,24]. Since many mental disorders begin in childhood or adolescents [46,47], children and adolescents are at severe risk of developing psychological distress and mental illness [47,48,49]. Given the enormous personal and societal burdens of mental illness, it might be profitable to begin mental health predictions and interventions in childhood. The development of mindfulness-related research may increase the chance of mindfulness practices, thus promoting children’s mental health.

In the current study, an optimal cutoff score of 22 or higher revealed a significant predictive power for subjective well-being, PYD, depression, and anxiety among children. Determining a valid cutoff point with significant predictive power is meaningful; thus, it is possible to classify the participants into a high or low level of mindfulness easily. However, it is important to note that very few studies on the CAMM cutoff score have been conducted in other countries. Therefore, the proposed cutoff score must be interpreted with caution, and more studies on the cutoff score are needed to confirm the cutoff score’s predicting ability.

This study has some limitations that must be considered. Firstly, the participants originated from the general Chinese population, so the cohort may have contained people without clinical depression or anxiety, which may have limited the reliability of the result of the cutoff score. Future studies can carry out similar investigations among both the general population and patients with clinical depression or anxiety to provide further evidence in order to confirm an optimal cutoff score. Moreover, only certain types of reliability and validity analyses were performed in the current study. It is necessary to investigate the cross-time stability of the scale. Despite these limitations, this study is the first to examine the construct validity of the CAMM using an ESEM framework, and it identified an optimal cutoff score among children, which provides a reference value for future studies investigating the effect of multiple mindfulness measurement tools and interventions. In short, the Chinese version of the CAMM, with satisfactory psychometric properties, is suitable for primary school students in China.

## Figures and Tables

**Table 1:** Participant characteristics and reliabilities of measurements.

Characteristics	*n*	%		
Gender				
Male	670	52.2		
Female	613	47.8		
Grade				
Grade 5	645	50.3		
Grade 6	638	49.7		
Sibling				
Only child	329	25.6		
Non-only child	954	74.4		
Paternal education				
Junior middle school or below	264	20.6		
High school or equivalent	341	26.6		
Bachelor or equivalent	479	37.3		
Master or above	47	3.7		
Unclear	152	11.8		
Maternal education				
Junior middle school or below	320	24.9		
High school or equivalent	329	25.6		
Bachelor or equivalent	468	36.5		
Master or above	32	2.5		
Unclear	134	10.4		
**Measurements**	**M**	**SD**	**α**	**ω**
WHO-5	21.11	6.65	0.935	0.936
PYD-VSF	59.37	10.69	0.906	0.909
PHQ-9	4.12	4.97	0.911	0.907
GAD-7	2.69	4.12	0.931	0.931

Note. *n*: number of subjects. M: total mean score. SD: standard deviation of total score. α: Cronbach’s α. ω: McDonald’s ω. WHO-5: The World Health Organization—Five Well-being Index. PYD-VSF: the Five Cs of Positive Youth Development–Very Short Form. PHQ-9: the 9-item Patient Health Questionnaire. GAD-7: the 7-item Generalized Anxiety Disorder Scale.

**Table 2:** Test of goodness of fit of the original and Chinese versions of the CAMM for children and adolescents.

	*χ* ^2^	*df*	TLI	CFI	RMSEA	SRMR
			≥0.90	≥0.90	≤0.08	≤0.08
The original English study by Greco and Bear, 2011 (*n* = 332)						
One-factor CFA	—	—	0.87	0.90	0.07	0.06
The Chinese study by Liu et al., 2019 (*n* = 309)						
One-factor CFA	205.75 **	35	0.72	0.78	0.13	0.08
Two-factor CFA	99.47 **	34	0.89	0.92	0.08	0.05
The current study (*n* = 1283)						
One-factor CFA	446.231 ***	35	0.808	0.850	0.096	0.066
One-factor ESEM	446.230 ***	35	0.808	0.850	0.096	0.066
Two-factor CFA	308.995 ***	34	0.867	0.900	0.079	0.056
Two-factor ESEM	168.251 ***	26	0.910	0.948	0.065	0.033

Note. ** *p* < 0.01; *** *p* < 0.001. *n*: number of subjects. CAMM: the Child and Adolescent Mindfulness Measure. CFA: Confirmatory Factor Analysis. ESEM: Exploratory Structural Equation Modeling. TLI: Tucker–Lewis Index. CFI: Bentler’s Comparative Fit Index. RMSEA: Root Mean Square Error of Approximation. SRMR: standardized root mean square residual.

**Table 3:** The correlation coefficients of the total scores and factor scores of the Chinese version of the CAMM and six criteria (*n* = 1283).

	Total	Factor 1	Factor 2
Subjective well-being	0.381 ***	0.482 ***	0.129 ***
PYD	0.287 ***	0.390 ***	0.060 *
Depression	−0.612 ***	−0.679 ***	−0.335 ***
Anxiety	−0.542 ***	−0.613 ***	−0.281 ***

Note. * *p* < 0.05; *** *p* < 0.001. PYD: positive youth development.

**Table 4:** Sensitivity, specificity, and Youden index for a selection of best cutoff points of the Chinese version of the CAMM for children.

	Subjective Well-Being			PYD			
Cutoff≥	Sensitivity	Specificity	Youden Index	Cutoff≥	Sensitivity	Specificity	Youden Index
20.5	0.8312	0.4182	0.2494	20.5	0.8906	0.2752	0.1658
21.5	0.7928	0.4727	0.2656	21.5	0.8587	0.3232	0.1819
*22.5*	*0.7460*	*0.5273*	*0.2732*	22.5	0.8252	0.3856	0.2108
23.5	0.7076	0.5455	0.2530	23.5	0.7948	0.4288	0.2236
24.5	0.6641	0.5909	0.2550	24.5	0.7538	0.4752	0.2290
25.5	0.6002	0.6182	0.2184	*25.5*	*0.6960*	*0.5392*	*0.2352*
26.5	0.5541	0.6636	0.2178	26.5	0.6429	0.5776	0.2205
	**Anxiety**			**Depression**			
Cutoff≥	Sensitivity	Specificity	Youden Index	Cutoff≥	Sensitivity	Specificity	Youden Index
20.5	0.8467	0.7229	0.5696	20.5	0.8758	0.6959	0.5717
21.5	0.8075	0.7711	0.5786	*21.5*	*0.8388*	*0.7568*	*0.5955*
*22.5*	*0.7600*	*0.8193*	*0.5793*	22.5	0.7912	0.8041	0.5952
23.5	0.7217	0.8313	0.5530	23.5	0.7507	0.8108	0.5615
24.5	0.6750	0.8313	0.5063	24.5	0.7048	0.8378	0.5427
25.5	0.6142	0.8916	0.5057	25.5	0.6414	0.8784	0.5198
26.5	0.5658	0.9036	0.4694	26.5	0.5912	0.8919	0.4831

Note. Estimates in italic typeface are the suggested optimal cutoffs. PYD: positive youth development.

## Data Availability

The data presented in this study are available on request from the corresponding author.

## References

[1] Mahinda V. (2009). Note of Abhidhamma (No.1).

[2] Kabat-Zinn J. (2003). Mindfulness-based interventions in context: Past, present, and future. Clin. Psychol. Sci. Pract..

[3] Baer R.A. (2003). Mindfulness training as a clinical intervention: A conceptual and empirical review. Clin. Psychol. Sci. Pract..

[4] Greco L.A., Baer R.A., Smith G.T. (2011). Assessing mindfulness in children and adolescents: Development and validation of the Child and Adolescent Mindfulness Measure (CAMM). Psychol. Assess..

[5] Bruin E.I., Zijlstra B.J., Bögels S.M. (2013). The meaning of mindfulness in children and adolescents: Further validation of the Child and Adolescent Mindfulness Measure (CAMM) in two independent samples from the Netherlands. Mindfulness.

[6] Kuby A.K., McLean N., Allen K. (2015). Validation of the Child and Adolescent Mindfulness Measure (CAMM) with non-clinical adolescents. Mindfulness.

[7] Viñas F., Malo S., González M., Navarro D., Casas F. (2015). Assessing mindfulness on a sample of Catalan-speaking Spanish adolescents: Validation of the Catalan version of the child and adolescent mindfulness measure. Span. J. Psychol..

[8] García-Rubio C., Rodríguez-Carvajal R., Langer A.I., Paniagua D., Steinebach P., Andreu C.I., Vara M.D., Cebolla A. (2019). Validation of the Spanish version of the child and adolescent mindfulness measure (CAMM) with samples of Spanish and Chilean children and adolescents. Mindfulness.

[9] Chiesi F., Dellagiulia A., Lionetti F., Bianchi G., Primi C. (2016). Using item response theory to explore the psychometric properties of the Italian version of the Child and Adolescent Mindfulness Measure (CAMM). Mindfulness.

[10] Dion J., Paquette L., Daigneault I., Godbout N., Hébert M. (2017). Validation of the French version of the Child and Adolescent Mindfulness Measure (CAMM) among samples of French and indigenous youth. Mindfulness.

[11] Sünbül Z.A. (2018). Psychometric Evaluation of Child and Adolescent Mindfulness Measure (CAMM) with Turkish Sample. Online Submiss..

[12] Roux B., Franckx A.C., Lahaye M., Deplus S., Philippot P. (2019). A french validation of the child and adolescent mindfulness measure (CAMM). Eur. Rev. Appl. Psychol..

[13] Mohsenabadi H., Shabani M.J., Assarian F., Zanjani Z. (2020). Psychometric properties of the child and adolescent mindfulness measure: A psychological measure of mindfulness in youth. Iran. J. Psychiatry Behav. Sci..

[14] Theofanous A., Ioannou M., Zacharia M., Georgiou S.N., Karekla M. (2020). Gender, age, and time invariance of the child and adolescent mindfulness measure (CAMM) and psychometric properties in three Greek-speaking youth samples. Mindfulness.

[15] Liu X., Chi X., Zhang J., Duan W., Wen Z. (2019). Validation of Child and adolescent Mindfulness Measure (CAMM) in Chinese Adolescents. Psychol. Explor..

[16] Asparouhov T., Muthén B. (2009). Exploratory structural equation modeling. Struct. Equ. Model. Multidiscip. J..

[17] Campion J., Rocco S. (2009). Minding the mind: The effects and potential of a school-based meditation programme for mental health promotion. Adv. Sch. Ment. Health Promot..

[18] Mendelson T., Greenberg M.T., Dariotis J.K., Gould L.F., Rhoades B.L., Leaf P.J. (2010). Feasibility and preliminary outcomes of a school-based mindfulness intervention for urban youth. J. Abnorm. Child Psychol..

[19] Gao L., Geng Y., Liu X. (2014). Mindfulness and Subjective Well-being among Junior School Students: Mediating Role of Self-esteem. China J. Health Psychol..

[20] Bluth K., Eisenlohr-Moul T.A. (2017). Response to a mindful self-compassion intervention in teens: A within-person association of mindfulness, self-compassion, and emotional well-being outcomes. J. Adolesc..

[21] Schonert-Reichl K.A., Oberle E., Lawlor M.S., Abbott D., Thomson K., Oberlander T.F., Diamond A. (2015). Enhancing cognitive and social–emotional development through a simple-to-administer mindfulness-based school program for elementary school children: A randomized controlled trial. Dev. Psychol..

[22] Rodríguez-Ledo C., Orejudo S., Cardoso M.J., Balaguer Á., Zarza-Alzugaray J. (2018). Emotional intelligence and mindfulness: Relation and enhancement in the classroom with adolescents. Front. Psychol..

[23] Semple R.J., Lee J., Rosa D., Miller L.F. (2010). A randomized trial of mindfulness-based cognitive therapy for children: Promoting mindful attention to enhance social-emotional resiliency in children. J. Child Fam. Stud..

[24] Lu S., Huang C.C., Rios J. (2017). Mindfulness and academic performance: An example of migrant children in China. Child. Youth Serv. Rev..

[25] Oberle E., Schonert-Reichl K.A., Lawlor M.S., Thomson K.C. (2012). Mindfulness and Inhibitory Control in Early Adolescence. J. Early Adolesc..

[26] Singh N.N., Lancioni G.E., Singh Joy S.D., Winton A.S., Sabaawi M., Wahler R.G., Singh J. (2007). Adolescents with conduct disorder can be mindful of their aggressive behavior. J. Emot. Behav. Disord..

[27] Lee J., Semple R.J., Rosa D., Miller L. (2008). Mindfulness-based cognitive therapy for children: Results of a pilot study. J. Cogn. Psychother..

[28] Haydicky J., Wiener J., Badali P., Milligan K., Ducharme J.M. (2012). Evaluation of a mindfulness-based intervention for adolescents with learning disabilities and co-occurring ADHD and anxiety. Mindfulness.

[29] Allgaier A., Pietsch K., Frühe B., Prast E., Sigl-Glöckner J., Schulte-Körne G. (2012). Depression in pediatric care: Is the WHO-Five Well-Being Index a valid screening instrument for children and adolescents?. Gen. Hosp. Psychiatry.

[30] Geldhof G.J., Bowers E.P., Boyd M.J., Mueller M.K., Napolitano C.M., Schmid K.L., Lerner J.V., Lerner R.M. (2014). Creation of short and very short measures of the five Cs of positive youth development. J. Res. Adolesc..

[31] Huang L., Liang K., Chen S., Kang W., Chi X. (2022). Validity and reliability of the Chinese version of the 5Cs Positive Youth Development Scale—Very Short Form. Chin. Ment. Health J..

[32] Bian C., He X., Qian J., Wu W., Li C. (2009). The reliability and validity of a modified patient health questionnaire fore screening depressive syndrome in general hospital outpatients. J. Tongji Univ. (Med. Sci.).

[33] Hu X., Zhang Y., Liang W., Zhang H., Yang S. (2014). Reliability and validity of patient health questionnaire Depression Scale (PHQ-9) in adolescents. Sichuan Ment. Health.

[34] Spitzer R.L., Kroenke K., Williams J.B., Löwe B. (2006). A brief measure for assessing generalized anxiety disorder: The GAD-7. Arch. Intern. Med..

[35] He X., Li C., Qian J., Cui H., Wu W. (2010). Reliability and validity of generalized anxiety disorder scale in general hospital outpatients. Shanghai Arch. Psychiatry.

[36] Browne M.W. (2001). An Overview of Analytic Rotation in Exploratory Factor Analysis. Multivar. Behav. Res..

[37] Hu L.T., Bentler P.M. (1999). Cutoff criteria for fit indexes in covariance structure analysis: Conventional criteria versus new alternatives. Struct. Equ. Model. Multidiscip. J..

[38] Wen Z., Hau K.T., Herbert W.M. (2004). Strucutal equation model testing: Cutoff criteria for goodness of fit indices and Chi-square test. Acta Psychol. Sin..

[39] Youden W.J. (1950). Index for rating diagnostic tests. Cancer.

[40] Baer R.A., Smith G.T., Allen K.B. (2004). Assessment of mindfulness by self-report: The Kentucky Inventory of Mindfulness Skills. Assessment.

[41] Duan W. (2014). Disagreements of Studies on Mindfulness: Conceptualization and Measurements. Adv. Psychol. Sci..

[42] Bishop S.R., Lau M., Shapiro S., Carlson L., Anderson N.D., Carmody J., Segal Z.V., Abbey S., Speca M., Devins G. (2004). Mindfulness: A proposed operational definition. Clin. Psychol. Sci. Pract..

[43] Neff K.D., Tóth-Király I., Yarnell L.M., Arimitsu K., Castilho P., Ghorbani N., Guo H.X., Hirsch J.K., Hupfeld J., Hutz C.S. (2019). Examining the factor structure of the Self-Compassion Scale in 20 diverse samples: Support for use of a total score and six subscale scores. Psychol. Assess..

[44] Mai Y., Wen Z. (2014). Exploratory Structural Equation Modeling (ESEM): An integration of EFA and CFA. Adv. Psychol. Sci..

[45] Asparouhov T., Muthén B., Morin A.J.S. (2015). Bayesian structural equation modeling with cross-loadings and residual covariances: Comments on Stromeyer et al. J. Manag..

[46] Kessler R.C., Angermeyer M., Anthony J.C., DE Graaf R., Demyttenaere K., Gasquet I., DE Girolamo G., Gluzman S., Gureje O., Haro J.M. (2007). Lifetime prevalence and age-of-onset distributions of mental disorders in the World Health Organization’s World Mental Health Survey Initiative. World Psychiatry.

[47] Patton G.C., Coffey C., Romaniuk H., Mackinnon A., Carlin J.B., Degenhardt L., Olsson C.A., Moran P. (2014). The prognosis of common mental disorders in adolescents: A 14-year prospective cohort study. Lancet.

[48] Hou J., Chen Z. (2021). The Interannual Evolution of Adolescents’ Mental Health Status in 2009 and 2020.

[49] Zhou H., Li D., Song Y., Zong C., Wu J., Lu H. (2007). Epidemiologic study of anexiety state in adolescents in China. J. Shanghai Jiaotong Univ. (Med. Sci.).

